# MicroCT Enables Simultaneous Longitudinal Tracking of Murine Pancreatic Cancer Progression and Cachexia

**DOI:** 10.1158/2767-9764.CRC-25-0414

**Published:** 2025-12-22

**Authors:** Katherine R. Pelz, Philip Jimenez, Colin J. Daniel, Samuel D. Newton, Melissa Cunningham, Rosalie C. Sears, Patrick J. Worth, Jonathan R. Brody, Teresa A. Zimmers

**Affiliations:** 1 Department of Biomedical Engineering, Oregon Health & Science University, Portland, Oregon.; 2 Brenden-Colson Center for Pancreatic Care, Oregon Health & Science University, Portland, Oregon.; 3 Department of Surgery, Oregon Health & Science University, Portland, Oregon.; 4 Department of Molecular and Medical Genetics, Oregon Health & Science University, Portland, Oregon.; 5 Department of Pediatrics, Oregon Health & Science University, Portland, Oregon.; 6 Department of Cell, Developmental and Cancer Biology, School of Medicine, Oregon Health & Science University, Portland, Oregon.; 7 Knight Cancer Institute, Oregon Health & Science University, Portland, Oregon.

## Abstract

Preclinical models of pancreatic ductal adenocarcinoma (PDAC) can greatly benefit from noninvasive imaging for evaluating disease progression and therapeutic response. Imaging approaches that can accurately and simultaneously track primary tumor growth, metastatic dissemination, and host cachexia over time are lacking. Herein, we report an optimized dual-contrast microCT protocol for longitudinal imaging in orthotopic murine models of PDAC. This method enables high-resolution, volumetric quantification of orthotopic primary tumors, liver and lung metastases, and paraspinal skeletal muscle, providing a dynamic view of both the tumor and host physiology. MicroCT primary tumor measurements were strongly correlated with endpoint tumor weights and outperformed 2D ultrasound in early detection and volumetric accuracy, particularly for small or irregularly shaped tumors. This platform revealed heterogeneous metastatic kinetics across PDAC models and uncovered an early, heterogeneous onset of skeletal muscle wasting, a hallmark of cancer cachexia. Notably, this protocol mimics clinical CT surveillance by enabling opportunistic cachexia assessment from tumor imaging datasets and offers substantial advantages over destructive endpoint analyses. Furthermore, microCT radiation dose had no effect on model endpoints. By capturing the temporal dynamics of tumor progression and host response, dual-contrast microCT serves as a powerful translational platform for preclinical PDAC research and therapeutic testing.

**Significance:**

Dual-contrast microCT provides high-resolution, whole-body, noninvasive imaging in orthotopic murine PDAC models, enabling simultaneous tracking of tumor growth, metastasis, and skeletal muscle wasting, offering a clinically relevant, translational imaging platform.

## Introduction

Pancreatic ductal adenocarcinoma (PDAC) remains one of the deadliest malignancies, with a poor prognosis due to treatment resistance, aggressive metastatic spread, and high prevalence and severity of cancer-associated cachexia (1–3). Preclinical studies of PDAC rely heavily on genetic and orthotopic mouse models (4); however, longitudinal monitoring of PDAC progression, including primary tumor growth, the timing of metastasis, and associated cachexia, remains challenging. Common imaging modalities used in murine PDAC models include bioluminescence imaging (BLI) and ultrasound (US; ref. 5). BLI provides a convenient readout of tumor burden but lacks spatial resolution, limiting its utility for accurate volumetric measurements and organ-specific detection of metastases (6). BLI provides no information about body composition, obviating its use in cachexia studies. US offers a higher resolution, avoids radiation exposure, and can be used for muscle measurements to assess cachexia severity. However, conventional two-dimensional (2D) US often assumes a spherical tumor geometry, which reduces the accuracy. 3D US improves volumetric accuracy for primary tumor measurements (7), however the effectiveness of US in detecting metastases is limited due to rib interference when imaging common metastatic sites, such as the liver and lungs. Together, these limitations underscore the need for a robust, high-resolution imaging approach capable of providing detailed longitudinal measurements of both tumor progression and host wasting.

To address this gap, we hypothesized that microCT imaging in conjunction with contrast administration could be used to longitudinally track pancreatic cancer progression in murine models, providing insights into the temporal dynamics of primary tumor growth, metastatic dissemination, and cachexia. MicroCT offers high spatial resolution and capacity for whole-body imaging, making it a promising tool for comprehensive tumor monitoring (Supplementary Fig. S1; ref. 8). However, to our knowledge, there are no protocols available describing the use of microCT for the concurrent monitoring of PDAC primary tumors, metastases, and cachexia.

Herein, we report an optimized dual-contrast microCT protocol for serial, noninvasive imaging of orthotopic pancreatic tumors. This method enables simultaneous, high-resolution tracking of primary tumor growth, metastasis, and concurrent muscle wasting, providing precise longitudinal data on PDAC and host dynamics, with minimal impact on animal health.

## Materials and Methods

### Animals

Male C57BL6/J mice (JAX, cat. #000664, RRID: IMSR_JAX:000664), aged 11 weeks at surgery (PDAC models), and female CD2F1 mice (C26 model, Charles River Laboratories, RRID: IMSR_CRL:033) were housed in pathogen-free conditions at 22.5°C and 12 hours light/12 hours dark cycles. The animals were provided *ad libitum* access to water and food (Rodent Diet 5001, Purina Mills). Animals were scanned at the designated days after tumor injection from 0800 to 1100 hours. Mice were monitored daily for health status, and humane endpoints were defined as tumor weight ≥10% mouse body weight or a Hickman score ≤3, based on body condition score, behavior, and appearance (9). Mice were excluded from the study if they exhibited signs of unrelated illness or surgical complications within 48 hours of tumor implantation. All other mice were included and monitored longitudinally. Mice were randomly assigned to tumor or PBS control groups using a random number generator, with all groups weight-matched and represented within each cage. Group sizes were determined based on observed variability in tumor progression within each PDAC cell line model; no formal power analysis was performed. All studies were conducted in accordance with the NIH Guide for the Care and Use of Laboratory Animals and were approved by the Institutional Animal Care and Use Committee (IACUC) of Oregon Health and Science University.

### Tumor models

Six murine PDAC cell lines were used: KPC 32908, KPC 7107, KPC 8060, KMC Z682, KMC Z693, and KMC Z696 (KPC– LSL-*Kras*^*G12D/+*^; LSL-*Tp53*^*R172H/+*^; *Pdx1-Cre*; KMC– LSL-*Kras*^*G12D/+*^; *Rosa26*-LSL^*Myc/Myc*^; *Ptf1a*^*CreERTM*^; bioRxiv 2025.07.14.664767). The KPC 7107 and KPC 8060 lines were kindly provided by Dr. Tony Hollingsworth (University of Nebraska Medical Center), and the KPC 32908 line was kindly provided by Dr. David Tuveson (University of Pennsylvania, derived from ref. 10). The KMC Z682, KMC Z693, and KMC Z696 lines were provided by R.C. Sears (Oregon Health and Science University). The KMC Z693 and Z696 lines were used to compare the primary tumor volume measurements derived from 2D US and microCT (Fig. 2E and F). Cell line authentication was performed by the originating laboratories. Our laboratory confirmed that the KPC 32908, KPC 7107, KMC Z682, KMC Z693, and KMC Z696 lines are transcriptionally distinct based on their bulk RNA sequencing profiles. The KPC 7107 and 8060 lines tested negative for *Mycoplasma* in November 2023 (*Mycoplasma* Genus PCR; Charles River Research Animal Diagnostic Services, RRID: SCR_003792). The KPC 32908 line tested negative for infectious diseases, including *Mycoplasma*, in July 2023 (OHSU Rodent CLEAR PCR Panel; Charles River Research Animal Diagnostic Services, RRID: SCR_003792). All six lines were additionally tested in-laboratory for *Mycoplasma* every 3 months during experimental use (SouthernBiotech, cat. #13100-01), and all tests were negative. All cell lines were cultured in DMEM (Thermo Fisher Scientific, cat. #11995065) supplemented with 10% FBS (Thermo Fisher Scientific, cat. #A5256801) and 1% penicillin–streptomycin (Thermo Fisher Scientific, cat. #15070063). Cells were used experimentally within three passages after thawing. *Orthotopic PDAC model*: Immediately prior to surgery, the mice were administered a flank subcutaneous injection of meloxicam (5 mg/kg). Under isoflurane anesthesia, a small surgical incision was made in the upper left quadrant of the abdomen, retracting the skin layers, fascia, and muscle wall to expose the pancreas. The tail of the pancreas was injected with 5,000 cancer cells suspended in 20 μL of 1:1 Matrigel (Thermo Fisher Scientific, cat. #354234) and PBS (Thermo Fisher Scientific, cat. #14-190-250) or an equal volume of cell-free Matrigel and PBS. After wound closure, mice received a flank subcutaneous injection of 400 μL warm sterile PBS, and the wound area was covered with 2.5% bupivacaine hydrochloride. The mice received a second flank subcutaneous injection of meloxicam (5 mg/kg) 24 hours after surgery. A total of 5,000 cells were injected for each of the four PDAC cell lines. *Subcutaneous colon cancer model*: One million C26 tumor cells (NCI, RRID: CVCL_0240) were injected subcutaneously into the right flank under isoflurane anesthesia.

### Radiation versus US study

To assess the effects of radiation on primary tumor growth, metastasis, and cachexia, 5,000 KMC Z682 PDAC cells were orthotopically injected into 20 male C57BL6/J mice (JAX, cat. #000664, RRID: IMSR_JAX:000664). Mice were assigned to either the nonradiation (*N* = 10, US imaging) or radiation (*N* = 10, microCT imaging) group. Mice were imaged starting 15 days after injection, with scans performed weekly until day 29 and then every 4 days until the study endpoint (day 43). The time under anesthesia (isoflurane) and contrast administration (iohexol and VivoVist, following the protocol below) were matched between groups. At the endpoint, all mice underwent microCT imaging for volumetric metastatic burden assessment immediately before euthanasia and tissue harvesting. In total, two mice (two from the radiation cohort and zero from the nonradiation cohort) were euthanized early because of exceeding humane endpoints before the final time point and were excluded from endpoint analyses.

### MicroCT contrast administration

To visualize the primary tumor, iohexol (GE Healthcare, Omnipaque 300 mg I/mL) was diluted 1:3 in PBS and administered intraperitoneally at a final concentration of 1.2 mg iodine per gram of body weight immediately prior to microCT imaging. To enhance contrast between the liver tissue and metastases, a single intraperitoneal injection of 1.5 g/kg VivoVist (Nanoprobes #1301) was administered 24 hours before the first microCT scan.

### MicroCT imaging and reconstruction

Mice were imaged using a high-resolution SkyScan 1278 microCT scanner (Bruker, RRID: SCR_026204) 7 days after tumor cell injection and subsequently scanned every 3 to 4 days or as indicated. The scanner was equipped with a fully integrated physiologic monitor, which tracked temperature, breathing, and ECG during the scan, along with the cumulative radiation dose. Prior to each day of scanning, the microCT machine was aged by turning on the radiation for approximately 4 minutes, and then the flat-field correction was updated to ensure that the background was represented in a consistent gray level, controlling for interpixel intensity variations that would otherwise result in ring artifacts. Mice were scanned in the prone position to optimize paraspinal muscle measurements, as this orientation enhances muscle delineation from adjacent organs, such as the kidneys. Scans were performed at a 50 μm pixel size, 0.5-mm Al filter, 40 ms exposure time, rotation step of 0.250 degrees, and 180 degrees with no frame averaging. We gated our scans using a physiologic monitoring window, with thoracic movement as the trigger option. Image reconstruction was performed with a 20% beam hardening correction and ring artifact reduction of four using CT-Recon software (Bruker).

### Imaging data analysis

MicroCT reconstructions were analyzed using CTAn and CTVol software (Bruker) to quantify the primary tumor volume, metastatic burden, and paraspinal muscle area as well as to generate 3D renderings. The primary tumors were segmented, and their volumes were extracted using CTAn. For liver and lung metastases, each organ was manually scanned to identify individual lesions, and the total metastatic burden was calculated by summing the volumes of all the metastases. Given the predominantly spherical nature of these lesions, the metastatic volume was approximated using the diameter of the largest cross-sectional area:

$$Volume\;\left({mm}^{3}\right)\;=\;\;\frac{\pi \;\times \;d^{3}}{6}$$

d = diameter

The paraspinal muscle area was measured at the most rostral cross-section of the L5 vertebra and normalized to baseline values. Endpoint tumor and muscle weights were recorded postmortem to validate the imaging-based measurements.

For US imaging, the primary tumor volumes were estimated using the largest axial and sagittal cross-sectional areas by applying the following equations:

$${Cylinder}_{A}\;\left({mm}^{3}\right)\;=\;\frac{\left(H_{A1}+\;H_{A2}\right)\;}{2}\;\times \;A_{A}$$

$${Cylinder}_{S}\;\left({mm}^{3}\right)\;=\;H_{S}\times A_{S}$$

$$Spherical\;Volume\;\left({mm}^{3}\right)\;=\;\frac{\left({Cylinder}_{A}+\;{Cylinder}_{S}\right)\;}{2}\;\times \;\frac{2}{3}$$

H = height

A = area

$$X_{A}\;=X_{axial}$$

$$X_{S}\;=X_{sagittal}$$

Investigators were blinded to group allocation during image analysis.

### Histologic analysis

The tissues were harvested at the endpoint, fixed in 10% formalin for 24 hours at 4°C, and embedded in paraffin for histologic analysis. Hematoxylin and eosin (H&E) staining was used for tumor and metastasis characterization. IHC was performed using anti-HuR antibody (Proteintech, cat. #11910-1-AP, RRID: AB_11182183, rabbit, 1:400) and either 3,3′diaminobenzidine-conjugated (Cell Signaling Technology, cat. #8114, RRID: AB_10544930, goat anti-rabbit) or alkaline phosphatase (AP)–conjugated (Vector Laboratories, cat. #MP-5401, RRID: AB_2336536; horse anti-rabbit antibody) secondary antibody, followed by chromogenic visualization.

### Statistical analysis and reproducibility

Statistical analyses were performed using GraphPad Prism version 9 (RRID: SCR_000306). Tissue weights across PDAC cell lines and longitudinal paraspinal muscle % areas were analyzed using one-way ANOVA, with cell line as the between-subjects factor (5 levels: PBS, KPC 8060, KPC 7107, KPC 32908, and KMC Z682). Dunnett multiple comparisons test was used to compare each group to the control (PBS). Assumptions of normality were assessed using the Shapiro–Wilk test. Time-to-metastasis was conducted using Kaplan–Meier curves with log-rank tests. Analyses assumed independence of survival times. Mice that did not develop detectable metastases by the moribund endpoint were censored. Censoring was assumed to be noninformative. Correlations between imaging-derived tumor volumes and endpoint tumor weights were assessed using Pearson correlation coefficient. Tumor and tissue weight comparisons between the radiation and nonradiation groups were done using unpaired two-tailed Student *t* tests. Normality of data was assessed using the Shapiro–Wilk test. Statistical significance was defined as *P* ≤ 0.05. In all studies, data represent biological replicates (*N*) and are presented as the mean ± SD.

## Results

### MicroCT enables accurate primary tumor volume quantification and growth kinetics in murine PDAC orthotopic models

**Figure 1.**
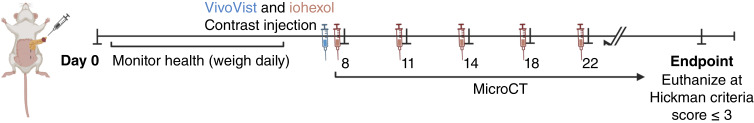
Study experimental timeline. Mice were orthotopically implanted with PDAC cells or PBS (day 0), injected with VivoVist (24 hours before the first scan) and iohexol (before each scan), and scanned every 3 to 4 days starting on day 8. Mice were euthanized upon reaching humane criteria (Hickman score ≤3; ref. 9).

To evaluate the sensitivity of microCT for preclinical imaging, we used a murine PDAC model with orthotopic injection of pancreatic tumor cells or PBS (control) and monitored the animals until they reached moribund status according to the IACUC guidelines [Hickman score ≤3 (9) or tumor weight ≥10% body weight; Fig. 1]. Based on prior optimization studies, 5,000 cells were injected to allow sufficient time for liver metastasis formation without early morbidity from the primary tumor. We tested four PDAC cell lines with distinct phenotypes and metastatic propensities, namely KPC 32908 (11, 12), KPC 7107, KPC 8060, and KMC Z682 (bioRxiv 2025.07.14.664767). KPC were derived from LSL-*Kras*^*G12D/+*^; LSL-*Tp53*^*R172H/+*^; *Pdx1-Cre* mice, and KMC from LSL-*Kras*^*G12D/+*^; *Rosa26*-LSL^*Myc/Myc*^; *Ptf1a*^*CreERTM*^ mice. Scans began 1 week after injection to allow for surgical recovery and was performed every 3 to 4 days (Fig. 1). The primary endpoints included longitudinal primary tumor volume, liver and lung metastasis volumes, skeletal muscle area, and latency to metastasis.

**Figure 2.**
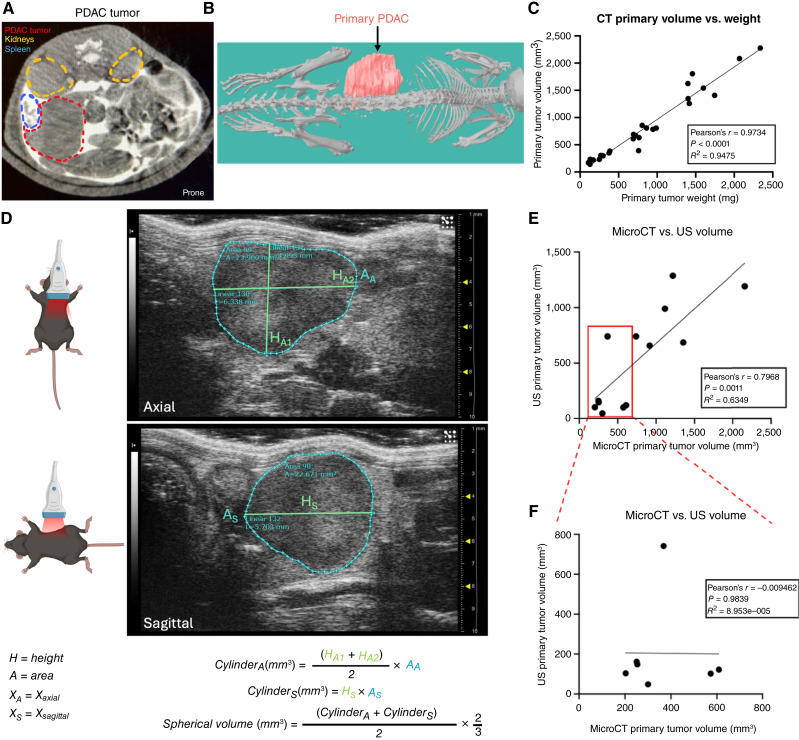
Longitudinal monitoring of PDAC tumor kinetics using microCT imaging and volumetric comparisons with 2D US. **A,** Representative axial microCT image showing the primary tumor (red outline). **B,** 3D reconstruction showing the primary tumor (red) and skeleton of the mouse rendered in grayscale. **C,** Correlation of primary tumor volume measured by microCT with tumor weight at endpoint (*N* = 27, Pearson’s *r* = 0.9734; *P* < 0.0001; *R*^2^ = 0.9475). **D,** Schematic and representative US images showing how primary tumor volume was estimated by 2D US. Axial and sagittal slices at the largest tumor dimensions were obtained, and area (A) and height (H) measurements were used to derive cylindrical volumes for each plane. Tumor volumes were calculated by averaging the volumes of the axial and sagittal cylinders and applying a spherical volume correction factor (2/3). **E,** Correlation between US- and microCT-derived primary tumor volumes (*R*^2^ = 0.6349, *P* = 0.0011). **F,** Correlation between US- and microCT-derived primary tumor volumes for subset of tumors smaller than 700 mm^3^ (as determined by microCT; *R*^2^ = 0.00, *P* = 0.9839).

To visualize PDAC tumors, iohexol (300 mg I/mL) diluted 1:3 in PBS (final concentration of 1.2 mg iodine per gram of body weight) was injected intraperitoneally immediately prior to each scan (Fig. 1), providing bright contrast throughout the peritoneal cavity to delineate the primary tumor from the surrounding organs (Fig. 2A; Supplementary Fig. S2). At this dose, iohexol was metabolized within 20 minutes (Supplementary Fig. S3). Using iohexol contrast with microCT imaging, we accurately quantified primary tumor volumes in our orthotopic PDAC model and generated 3D tumor images (Fig. 2B). MicroCT-derived volumes were strongly correlated with endpoint tumor weights (R^2^ = 0.9475; *P* < 0.0001; Fig. 2C). To validate microCT volumetric measurements against an established imaging method, we compared 2D US-derived tumor volumes (calculated from axial and sagittal areas using x-, y-, and z-coordinates; Fig. 2D) with endpoint tumor weights. US-derived volumes correlated less strongly with tumor weights than did microCT measurements (R^2^ = 0.5546; *P* = 0.0135; Supplementary Fig. S4). Direct comparison of tumor volumes obtained by microCT and US showed a moderate overall correlation (R^2^ = 0.6349; *P* = 0.0011; Fig. 2E); however, the correlation was lost for small tumors (<700 mm^3^ by microCT; R^2^ = 0.00; *P* = 0.9839; Fig. 2F), and US consistently underestimated volumes, likely due to the nonspherical tumor shape. These findings highlight that microCT is a superior imaging modality for accurate primary tumor measurement, particularly during early tumor progression. The accuracy of microCT tumor measurements was further demonstrated in subcutaneous C26 carcinoma models (Supplementary Fig. S5).

### MicroCT allows noninvasive, high-resolution tracking of PDAC liver and lung metastases

**Figure 3.**
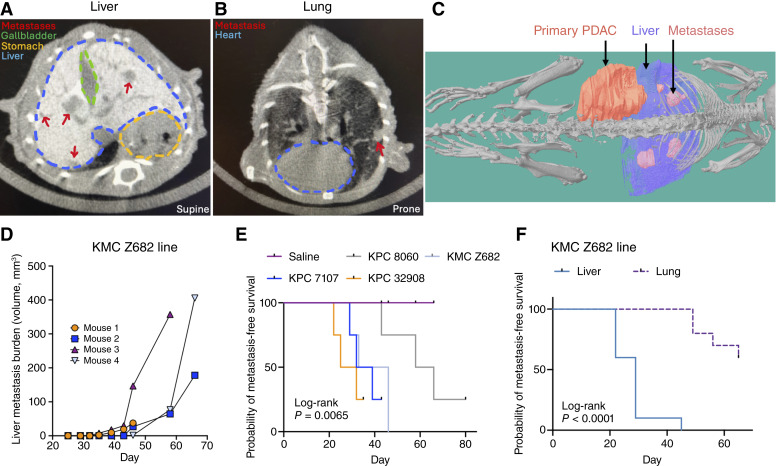
Longitudinal monitoring of PDAC liver and lung metastases using microCT imaging. **A** and **B,** Representative axial microCT images showing (**A**) liver metastases (red arrows) and (**B**) lung metastasis (red arrow). **C,** 3D reconstruction showing the primary tumor (red), liver (blue), metastases (pink), and skeleton of the mouse rendered in grayscale. **D,** Longitudinal liver metastatic burden in KMC Z682-injected mice (*N* = 4). **E,** Metastasis-free survival for PDAC lines (*N* = 4/group, *P* = 0.0065). **F,** Liver- vs. lung-specific metastasis-free survival in KMC Z682-injected mice (*N* = 10 mice, log-rank *P* < 0.0001).

To visualize PDAC liver metastases, a single intraperitoneal injection of 1.5 g/kg VivoVist (Nanoprobes #1301) was administered 24 hours before the first scan (Fig. 1), which enhanced the contrast between liver tissue and metastases (Fig. 3A). VivoVist, composed of alkaline earth metal nanoparticles, was rapidly taken up by the liver and spleen, maintaining consistent contrast intensity for the study duration (up to 72 days after injection; Supplementary Figs. S6–S8). Lung metastases require no additional contrast as air provides sufficient differentiation (Fig. 3B). A limitation of this protocol is the persistent contrast artifact from VivoVist in liver and spleen histopathology, presenting as a dark brown hue in both H&E and IHC staining (Supplementary Fig. S9). To mitigate this, we recommend immunofluorescence for protein analysis or alkaline phosphatase–conjugated antibodies for IHC, as red staining is distinguishable from brown artifacts when analyzed using software such as ImageJ (Supplementary Fig. S9).

MicroCT imaging with VivoVist contrast enabled precise, noninvasive tracking of metastasis progression (Fig. 3C; Supplementary Fig. S10). We focused on liver and lung metastases, which are the most common sites of metastasis in PDAC (10, 13, 14), however, metastases were also detected in the spleen in images and at euthanasia (Supplementary Fig. S11). Longitudinal monitoring allowed the volumetric quantification of liver metastases over time (Fig. 3D). Our orthotopic PDAC models exhibited substantial inter- and intra-line heterogeneity, emphasizing the importance of serial imaging for robust comparison. Longitudinal analyses improve sensitivity over single-time-point comparisons, enhancing statistical power through repeated-measures models rather than *t* tests at one time point. Kaplan–Meier analysis showed distinct metastatic timing among PDAC lines: KPC 32908 (28.5 days), KPC 7107 (35.5 days), KMC Z682 (39.5 days), and KPC 8060 (62 days; *P* = 0.0065, log-rank test, Fig. 3E). In the KMC Z682 model, mice frequently develop lung and liver metastases. MicroCT identified the emergence of liver metastases significantly earlier than lung metastases (median 29 vs. 52.5 days, *P* < 0.0001, log-rank test, Fig. 3F). Compared with US, microCT provided superior consistency and spatial resolution when tracking liver and lung metastases in longitudinal studies (Supplementary Table S1). The ability to quantify the metastatic burden volumetrically via microCT also offers distinct advantages over endpoint histologic or flow cytometry–based analyses, which require large cohort sizes and extensive tissue processing. Histology and flow cytometry require complete serial sectioning or tissue digestion to detect small lesions, whereas microCT noninvasively captures whole-organ metastasis.

### Longitudinal microCT imaging reveals early and heterogenous onset of cachexia-induced skeletal muscle wasting

**Figure 4.**
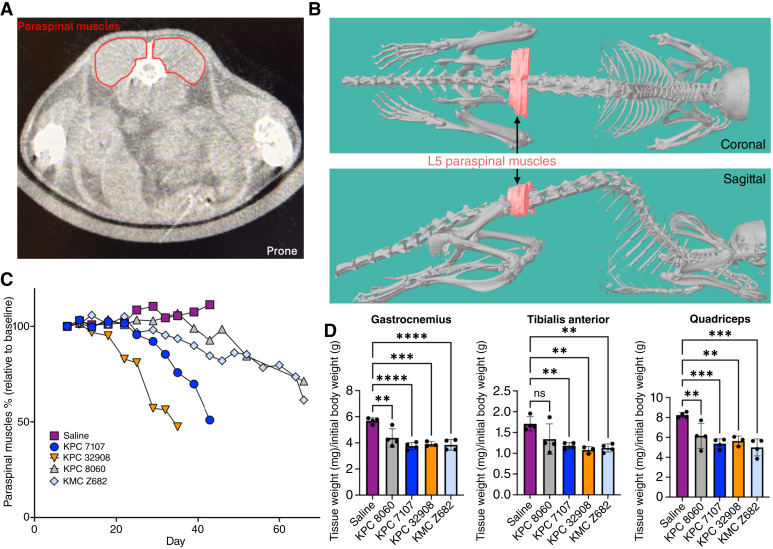
Longitudinal assessment of skeletal muscle wasting by microCT. **A,** Representative axial microCT image showing paraspinal muscle cross-section at the L5 vertebra (red outline)—the region used for area measurements. **B,** 3D reconstruction of microCT scan showing L5 paraspinal muscles (red) in the coronal and sagittal planes. **C,** Longitudinal tracking of paraspinal muscle area, expressed as a percentage of baseline for mice injected with different PDAC lines or PBS control. **D,** Postmortem analysis of gastrocnemius, tibialis anterior, and quadriceps muscle weights normalized to initial body weight across experimental groups (*N* = 4 per group). Statistical analysis used one-way ANOVA with Dunnett multiple comparisons test. ns, not significant; **, *P* < 0.01; ***, *P* < 0.001; ****, *P* < 0.0001. Error bars represent the mean ± SD.

Analogous to the opportunistic use of clinical diagnostic CT scans to quantify muscle loss, which is the current gold standard for monitoring cachexia in clinical studies, we tracked the paraspinal muscle area longitudinally (Fig. 4A and B). All PDAC cell lines exhibited significant muscle wasting. KPC 32908 produced the most rapid decline; the muscle area decreased by 43% by day 29, when all other lines were reduced by <10% (Fig. 4C). Significant decreases in the paraspinal muscle area were first detected on days 29 and 35 for KPC 32908 and 7107, respectively (Dunnett multiple comparisons test, *P* = 0.0210, *P* = 0.0188). However, paraspinal muscle weight showed a downward trend as early as days 18, 25, 35, and 39 for KPC 32908, KPC 7107, KMC Z682, and KPC 8060, respectively (Fig. 4C). All PDAC models showed signs of cachexia at the endpoint by standard measures, with hindlimb muscle weights normalized to initial body weight (30%–33% loss; Fig. 4D). The KPC 32908, KPC 7107, and KMC Z682 groups also had significantly reduced heart weights compared with the non-tumor bearing controls (Supplementary Fig. S12). These findings highlight both the prevalence of cachexia in PDAC and that skeletal muscle wasting is an early manifestation of the disease. Notably, different PDAC models demonstrated variable cachexia-inducing capacities at early time points. This heterogeneity is challenging to detect using endpoint analyses alone, highlighting the importance of longitudinal microCT imaging for capturing dynamic changes in muscle wasting and better understanding the progression of cachexia in preclinical cancer models.

### Imaging frequency considerations to balance data resolution with radiation exposure

**Figure 5.**
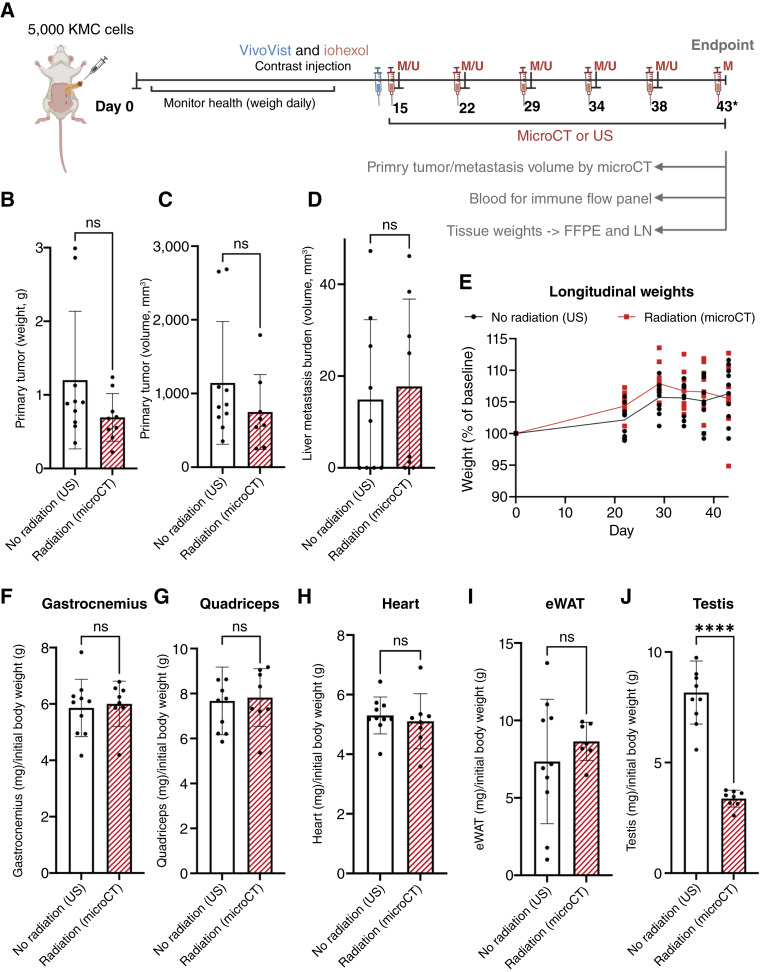
Radiation exposure effects on PDAC model tumorigenesis and muscle/adipose tissue weights. **A,** Experimental timeline: Mice were injected orthotopically with 5,000 KMC Z682 cells, and longitudinal imaging was performed via microCT (M) or US (U) at specified time points. All mice underwent microCT scanning immediately prior to euthanasia. Average radiation dose per scan was 715 mGy. **B,** Primary tumor weight (g) and (**C**) primary tumor and (**D**) liver metastasis volume (mm^3^) at euthanasia. **E,** Longitudinal body weight represented as % of baseline. **F–J,** Tissue weights normalized to initial body weight at euthanasia. **F,** Gastrocnemius, (**G**) quadriceps, (**H**) heart, (**I**) epididymal white adipose tissue (eWAT), and (**J**) testis. Statistical analysis was performed using *t* tests. ****, *P* < 0.0001, ns = not significant. Error bars represent the mean ± SD. FFPE, formalin-fixed, paraffin-embedded; LN, liquid nitrogen.

Of note, this approach parallels the clinical CT imaging used to track tumor progression in patients, in which scans typically occur every 3 months (15). Given the short lifespan of mice, our imaging frequency provided a comparable timescale for translational relevance. However, frequent scanning resulted in substantial radiation exposure of the mice (mean cumulative dose per mouse = 10 Gy over 66 days, Supplementary Table S2) and investigator time (4 minutes per scan and ∼15 minutes for reconstruction and analysis). Therefore, we recommend only using this scan frequency for initial cell line or model characterization, with reduced imaging intervals tailored to the desired experimental endpoints in subsequent studies. Although we did not observe overt radiation-induced effects on mouse health, distinguishing between radiation-specific and tumor-driven effects was challenging due to overlapping sickness behaviors. Comparisons between PBS control mice (receiving the same radiation regimen but without tumors) and PDAC-bearing mice indicated that the observed weight loss and muscle wasting were tumor-induced (Fig. 4D; Supplementary Fig. S13). To confirm that radiation had no effect on our model endpoints, we performed a study following our standard protocol (Fig. 1) but included a matched group that received contrast injections and anesthesia without microCT scanning (Fig. 5A). We found no differences in primary tumor weight or primary tumor and liver metastases volumes measured by microCT at euthanasia (day 43 after injection) between the groups (Fig. 5B–D), confirming that the radiation doses proposed in our protocol did not affect our model endpoints. Body weight trajectories were comparable between groups (Fig. 5E), as were skeletal muscle, heart, and epididymal adipose tissue weights at euthanasia (Fig. 5F–I). Radiation reduced testis size, which is consistent with previous reports (Fig. 5J; ref. 16). Regardless, careful consideration of cumulative radiation exposure is essential, and scan frequency should be optimized to balance the data collection needs and minimize radiation-related confounders. Indeed, in subsequent studies using the KMC Z682 line, we scanned weekly from day 15, increasing to every 3 days between days 29 and 46 to capture time-to-metastasis, and reverting to weekly scans thereafter to track tumor growth, metastasis volume, and skeletal muscle area.

## Discussion

In this study, we developed and validated a dual-contrast microCT protocol for preclinical monitoring of pancreatic cancer progression in murine models. By providing simultaneous, precise, longitudinal volumetric measurements of tumors, metastases to different organ sites, and cachexia-associated skeletal muscle wasting, our protocol surpasses conventional imaging methods like BLI and US (Supplementary Table S1), which often lack spatial resolution, suffer from signal attenuation due to tissue depth, or fail to simultaneously quantify tumor burden and host tissue changes.

To the best of our knowledge, this is the first study to describe the dual use of iohexol and VivoVist contrast agents for the simultaneous delineation of primary pancreatic tumors and liver metastases via microCT. Consistent with previous studies, we found that intraperitoneal administration of iohexol effectively enhanced soft tissue contrast in the peritoneal cavity, enabling precise localization of PDAC tumors and improved delineation from surrounding organs (17, 18). Whereas previous studies used fixed volumes of iohexol ranging from 250 μL to 3 mL, we found that injecting volumes closer to 100 μL of iohexol diluted 1:3 in PBS (to achieve a final concentration of 1.2 mg iodine per gram of body weight) effectively delineated the primary tumor while minimizing hyperdense artifacts that could compromise both primary tumor and paraspinal muscle volumetric measurements on CT imaging (19).

Our study also reports a novel application of VivoVist for the detection and volumetric tracking of liver metastases in preclinical cancer models. VivoVist, a nanoparticle-based contrast agent composed of alkaline earth metal cores, has been primarily described in the literature for intravenous use to enhance vascular imaging (20). In this study, we demonstrated that a single intraperitoneal dose of VivoVist (1.5 g/kg) enabled reliable distinction between liver parenchyma and metastatic lesions for the duration of our study, up to 72 days after injection. In contrast, hepatocyte-specific contrast agents such as 1,3-bis[7-(3-amino-2,4,6-triiodophenyl)heptanoyl]-2-oleoyl-glycerol have been shown to clear rapidly via the biliary system, limiting their window for effective tumor detection to within 24 hours after injection (21, 22). This dual-contrast protocol is broadly adaptable to other preclinical models involving abdominal tumors or liver metastasis, which are common in many solid tumors such as colorectal, pancreatic, and breast cancers (23).

Our dual-contrast microCT protocol was particularly advantageous during early tumor development and metastatic seeding, which are critical time points when conventional imaging modalities are lacking. US, which is widely used, has limited sensitivity for detecting small, irregularly shaped tumors or liver and lung metastases due to rib interference. BLI, although convenient for whole-body surveys, can produce ambiguous signals when multiple organs overlap or when tumors reside near high-background tissues, such as the liver or gut. Additionally, commonly used reporters such as luciferase and GFP have been shown to elicit immune responses in immunocompetent hosts (24). In contrast, our dual-contrast microCT protocol provided high-resolution, volumetric delineation of both primary and metastatic lesions, even in the early stages of the disease. This capability is especially important in pancreatic cancer, which is characterized by aggressive, early-onset metastatic spread (25), often before the primary tumor becomes symptomatic or radiographically apparent (26, 27). As a result, many patients present with advanced disease, limiting therapeutic options and contributing to poor survival outcomes. Preclinical models that fail to capture early metastatic events are unlikely to recapitulate the clinical trajectory of human PDAC and may overestimate the efficacy of investigational therapies. By enabling early visualization of metastases and their progression *in vivo*, our microCT protocol enhances mechanistic studies of metastatic seeding and organotropism and strengthens the translational value of preclinical therapeutic testing, particularly for agents targeting early dissemination or premetastatic niches.

Moreover, the capacity to longitudinally measure paraspinal skeletal muscle area in our study provided new insights into the kinetics of cachexia onset, an aspect of tumor biology that is often overlooked in preclinical models and is a major contributor to PDAC mortality (2, 28–31). We found that skeletal muscle loss occurred in a tumor line-dependent manner and could be detected earlier than would be appreciated by endpoint measurements alone. This finding highlights an underrecognized heterogeneity in cachexia progression and underscores the importance of dynamic and temporal assessments.

We explored the feasibility of quantifying hindlimb muscle areas using our microCT protocol, as the gastrocnemius, tibialis anterior, and quadriceps are conventional reference muscles in preclinical cachexia studies. However, these individual muscles are not clearly distinguishable by microCT at the imaging resolution and radiation dose used, precluding precise segmentation. To approximate total hindlimb muscle mass, we measured the cross-sectional area of the entire thigh musculature at the femoral midpoint but found variability due to differences in leg positioning between scans (Supplementary Fig. S14). Because the scan plane is not consistently perpendicular to the femoral axis, small changes in limb angle substantially affected the measured area, particularly as sick mice hunch and retract their limbs during late disease. In contrast, the spine can be consistently aligned along the imaging bed, and paraspinal muscle geometry is minimally affected by posture, providing a more reliable site for longitudinal quantification. Notably, this approach is analogous to the opportunistic use of clinical diagnostic CT scans to quantify muscle loss, in which skeletal muscle area at the third lumbar vertebra is used as a validated surrogate for total body muscle mass (32). Together, these features make paraspinal muscle quantification a technically robust and translationally relevant metric for studying cancer cachexia using our microCT platform.

This study used syngeneic rather than patient-derived xenograft (PDX) PDAC models to preserve immunocompetent tumor–host interactions. An intact immune system is important for studying metastatic progression and cancer-associated cachexia, which are increasingly recognized as being shaped by immune-mediated mechanisms, including systemic inflammation and metabolic dysregulation (33, 34). However, we anticipate that our dual-contrast microCT imaging protocol is broadly applicable across preclinical PDAC models. The ability to noninvasively perform high-resolution, volumetric quantification of orthotopic and subcutaneous (see C26 colon model data, Supplementary Fig. S5) primary tumors, liver and lung metastases, and paraspinal skeletal muscle should be preserved in PDX and organoid-derived systems.

Although frequent imaging improves resolution and statistical power by reducing the number of animals required, we recognize that cumulative radiation exposure and imaging demands are nontrivial. However, control experiments supported that radiation effects did not affect our study endpoints, such as primary tumor growth, metastasis burden, or cachexia. Future applications of this protocol may benefit from optimizing scan frequency based on the experimental phase, for example, increasing frequency during early metastatic seeding or cachexia onset, while decreasing frequency during stable tumor phases–to balance information yield with animal welfare and resource constraints.

Taken together, our findings establish dual-contrast microCT as a robust and versatile platform for preclinical cancer research. By enabling the integrated assessment of primary tumor growth, metastatic dissemination to multiple organs, and systemic wasting over time, this imaging protocol expands the toolkit for studying complex cancer phenotypes and evaluating therapeutic interventions in preclinical models.

## Supplementary Material

Supplementary Figure 1 MicroCT workflow

Supplementary Figure 2 Biodistribution of iohexol contrast injected intraperitoneally

Supplementary Figure 3 Time course of iohexol contrast distribution following intraperitoneal injection visualized by microCT imaging

Supplementary Figure 4 Correlation between ultrasound-derived tumor volumes and endpoint tumor weights in orthotopic PDAC model

Supplementary Figure 5 MicroCT 3D reconstruction of subcutaneous tumor from C26 colon cancer model

Supplementary Figure 6 Biodistribution of Vivovist contrast

Supplementary Figure 7 Longitudinal retention of Vivovist contrast visualized by microCT imaging

Supplementary Figure 8 Vivovist biodistribution visualized by hematoxylin and eosin (H&E) staining

Supplementary Figure 9 Vivovist contrast artifacts disrupt immunohistochemistry visualization and strategies to mitigate interference

Supplementary Figure 10 MicroCT-detected liver lesions correspond to histologically confirmed metastases

Supplementary Figure 11 MicroCT slice demonstrating a metastasis in the spleen

Supplementary Figure 12 Tumor-induced wasting of heart tissue

Supplementary Figure 13 Tumor-induced weight loss is independent of radiation dose

Supplementary Figure 14 Assessment of hindlimb muscle quantification using microCT

Supplementary Table 1 Imaging modality recommendations based upon primary endpoint

Supplementary Table 2 Cumulative radiation dose in reported study

## Data Availability

Data were generated by the authors and are available on request. No code was developed or used in this study.
