# Supplementary figures and images for: MicroCT Enables Simultaneous Longitudinal Tracking of Murine Pancreatic Cancer Progression and Cachexia
Source: Cancer Res Commun. 2025 Dec 22;5(12):2197–206. doi: 10.1158/2767-9764.CRC-25-0414 (PMC12719914; doi:10.1158/2767-9764.CRC-25-0414)

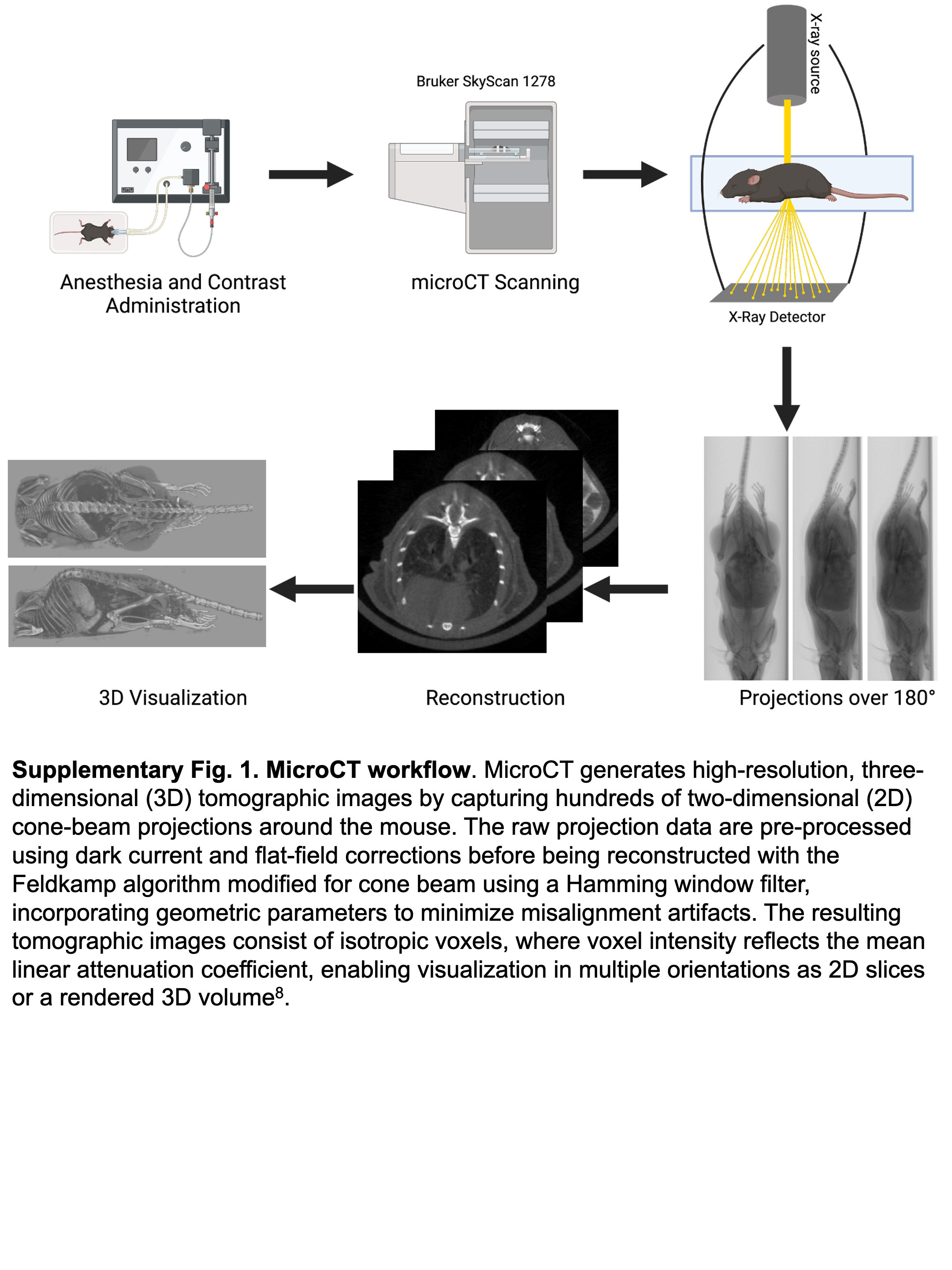

Supplement: Supplementary Figure 1 — MicroCT workflow [file crc-25-0414_supplementary_figure_1_suppsf1.png]

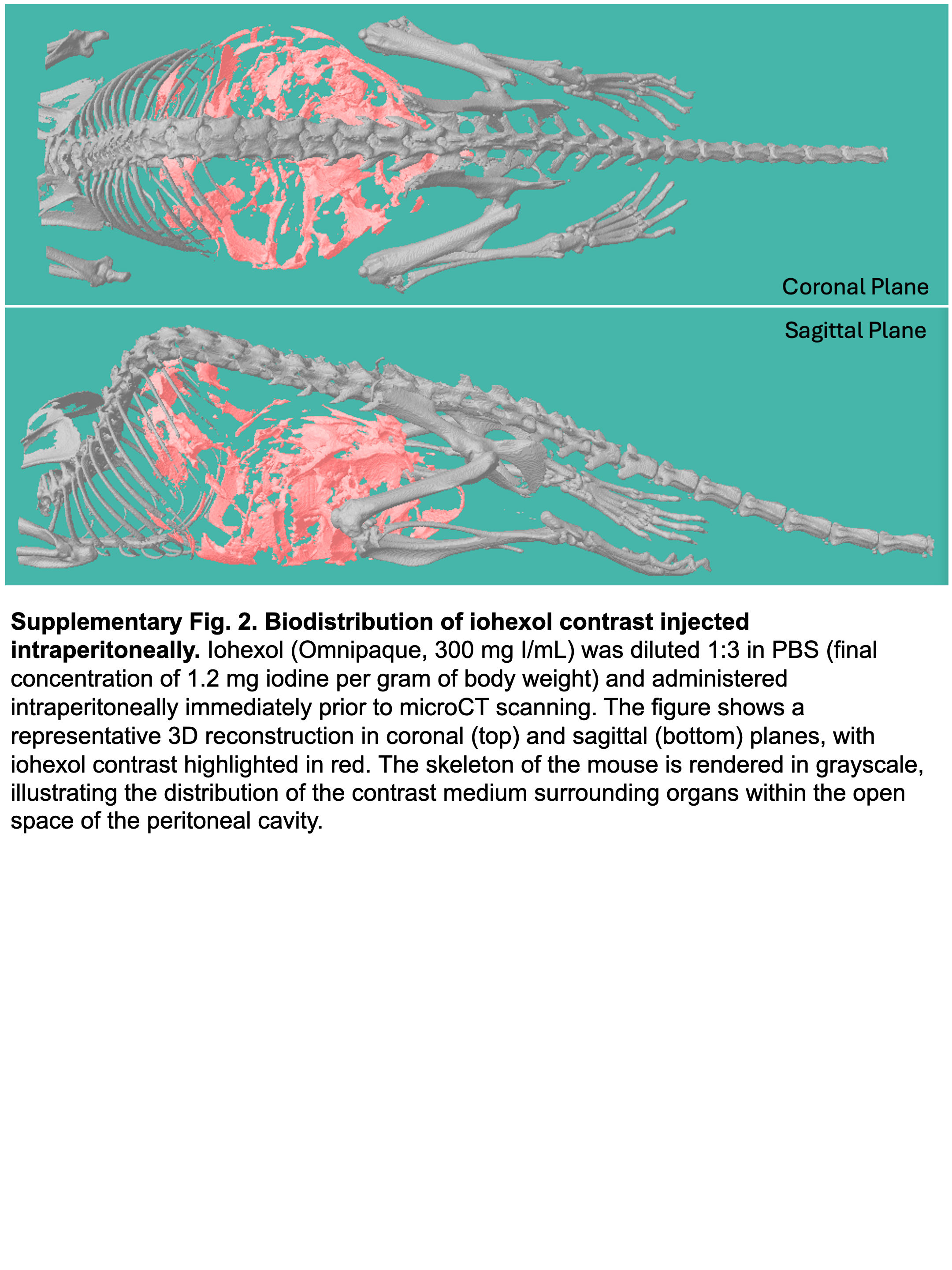

Supplement: Supplementary Figure 2 — Biodistribution of iohexol contrast injected intraperitoneally [file crc-25-0414_supplementary_figure_2_suppsf2.png]

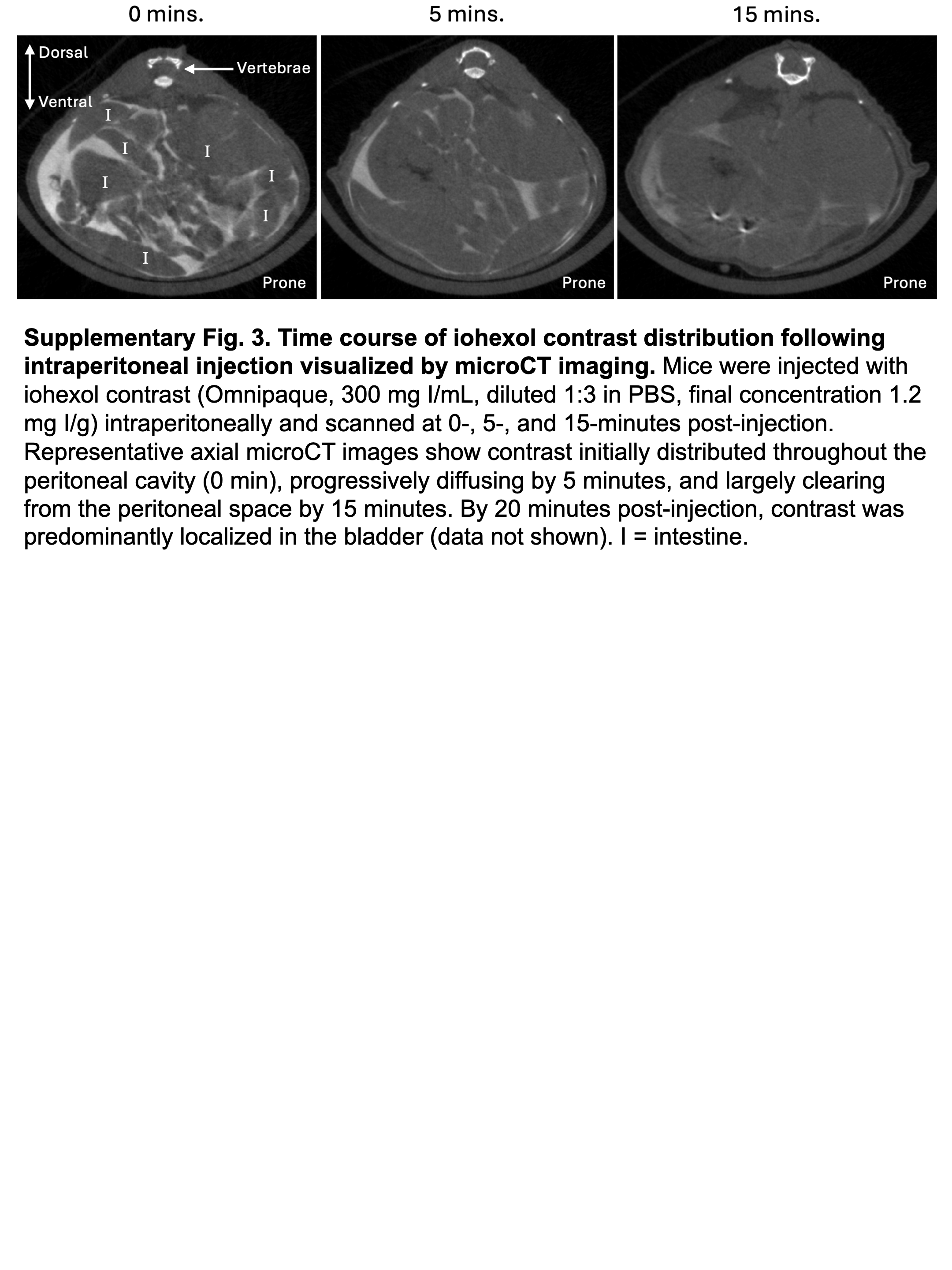

Supplement: Supplementary Figure 3 — Time course of iohexol contrast distribution following intraperitoneal injection visualized by microCT imaging [file crc-25-0414_supplementary_figure_3_suppsf3.png]

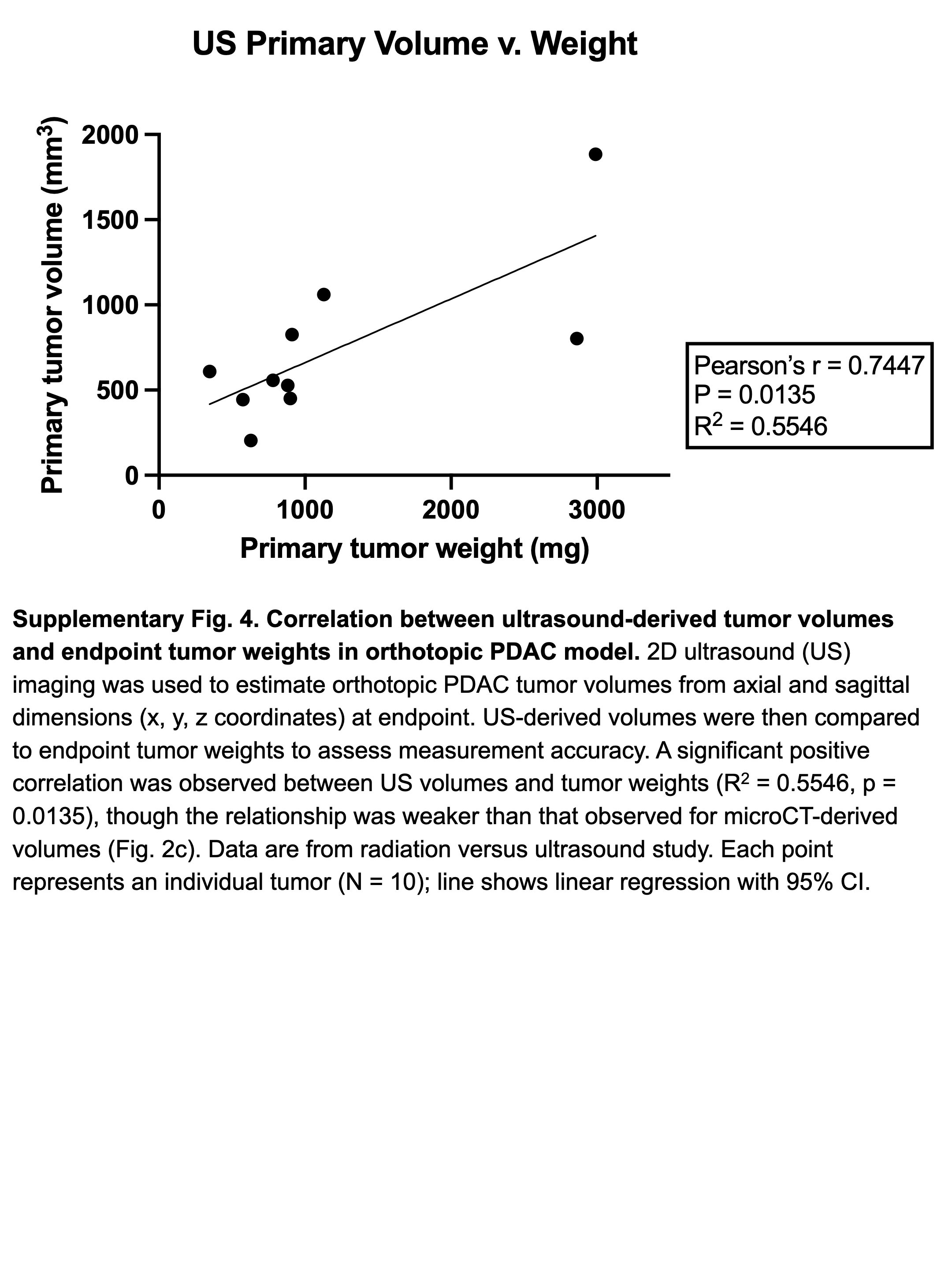

Supplement: Supplementary Figure 4 — Correlation between ultrasound-derived tumor volumes and endpoint tumor weights in orthotopic PDAC model [file crc-25-0414_supplementary_figure_4_suppsf4.png]

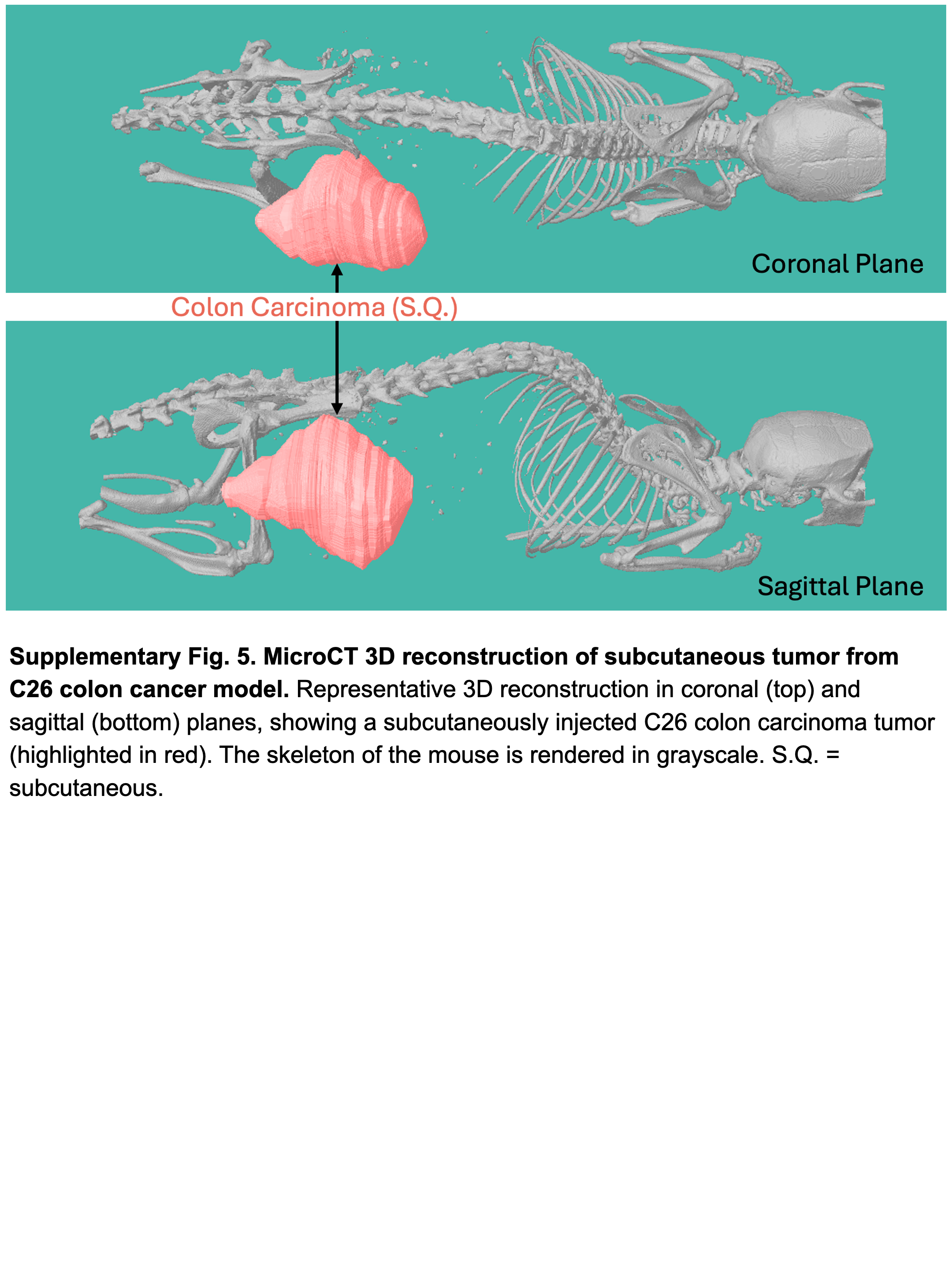

Supplement: Supplementary Figure 5 — MicroCT 3D reconstruction of subcutaneous tumor from C26 colon cancer model [file crc-25-0414_supplementary_figure_5_suppsf5.png]

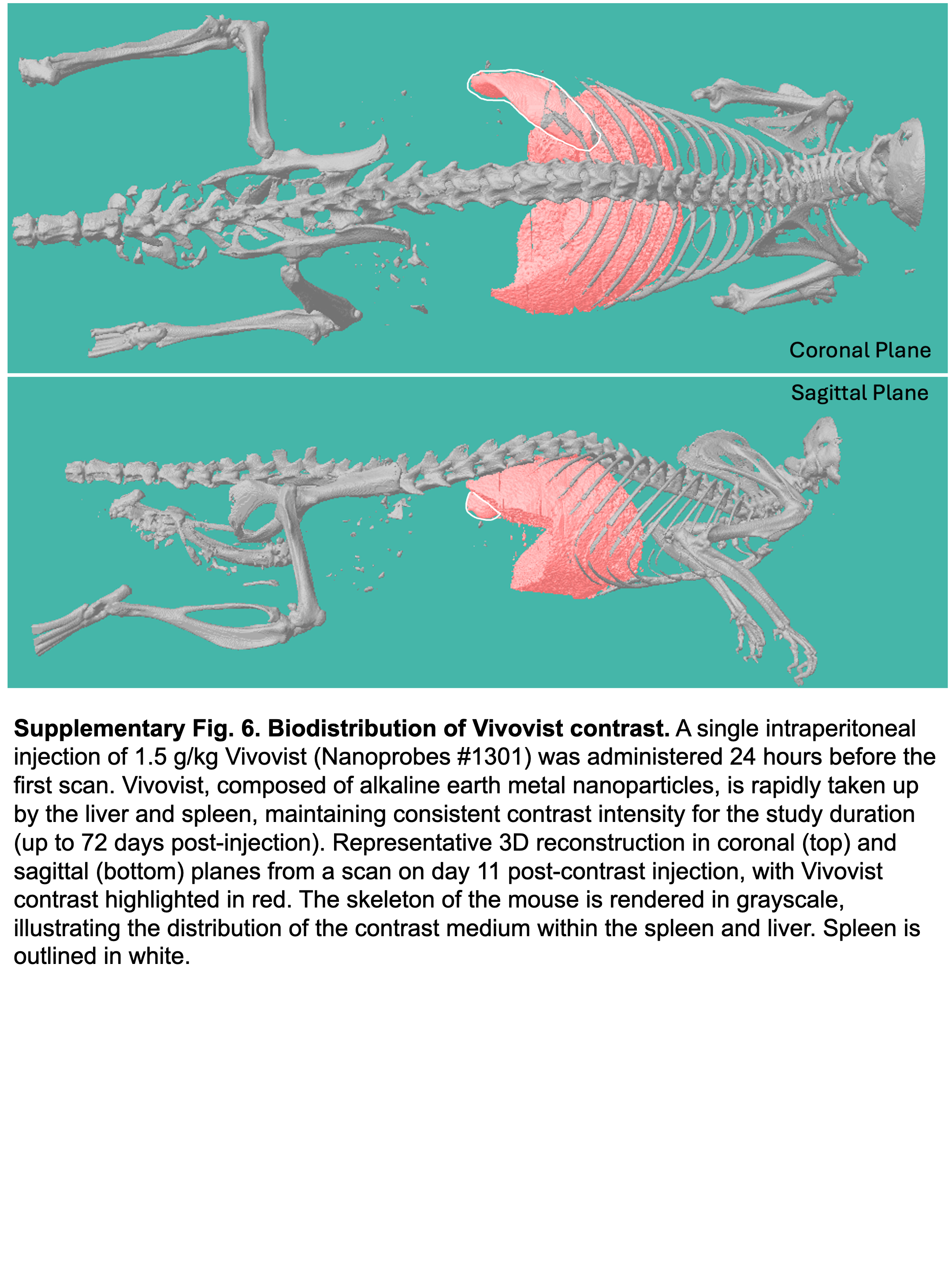

Supplement: Supplementary Figure 6 — Biodistribution of Vivovist contrast [file crc-25-0414_supplementary_figure_6_suppsf6.png]

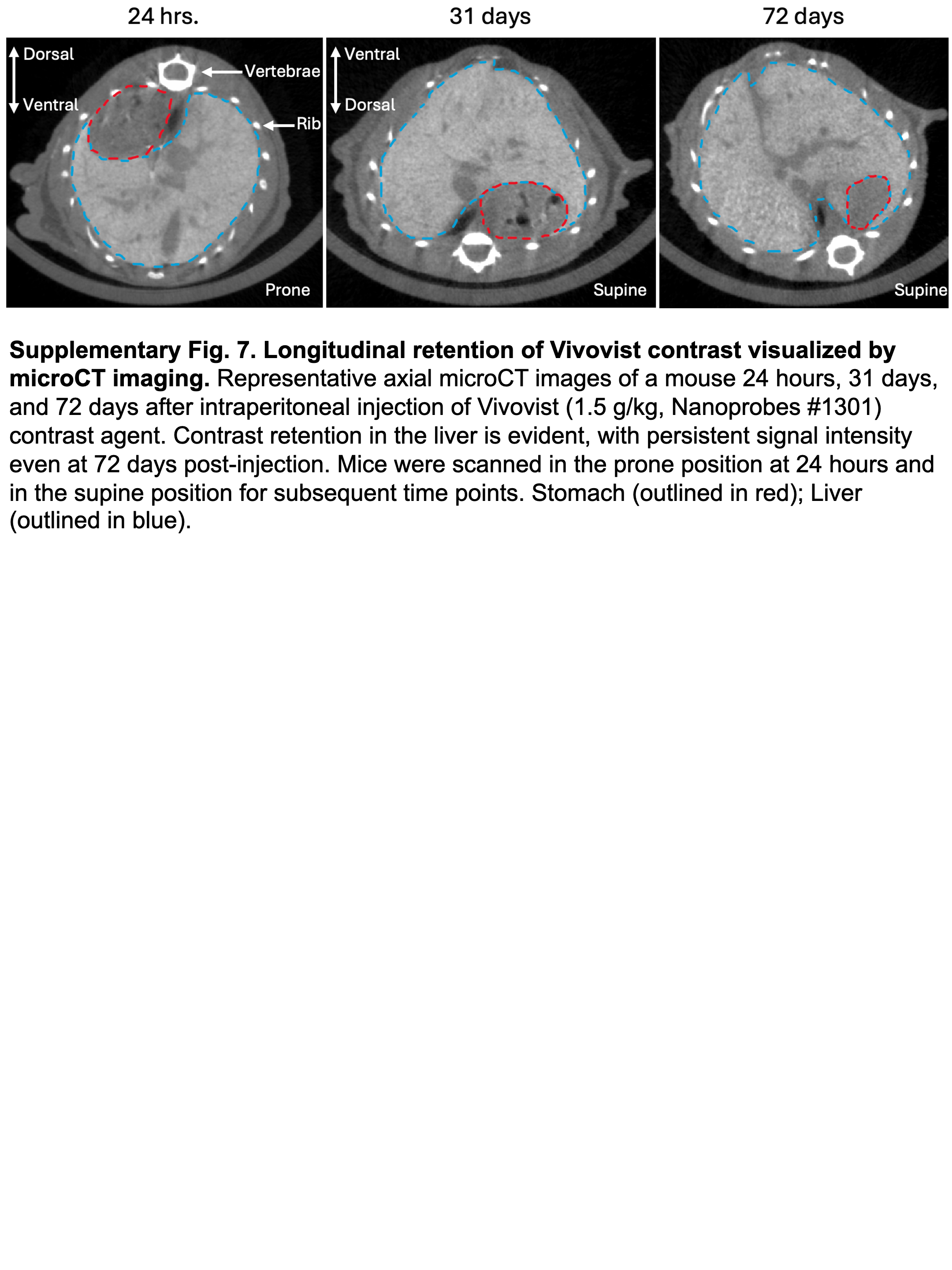

Supplement: Supplementary Figure 7 — Longitudinal retention of Vivovist contrast visualized by microCT imaging [file crc-25-0414_supplementary_figure_7_suppsf7.png]

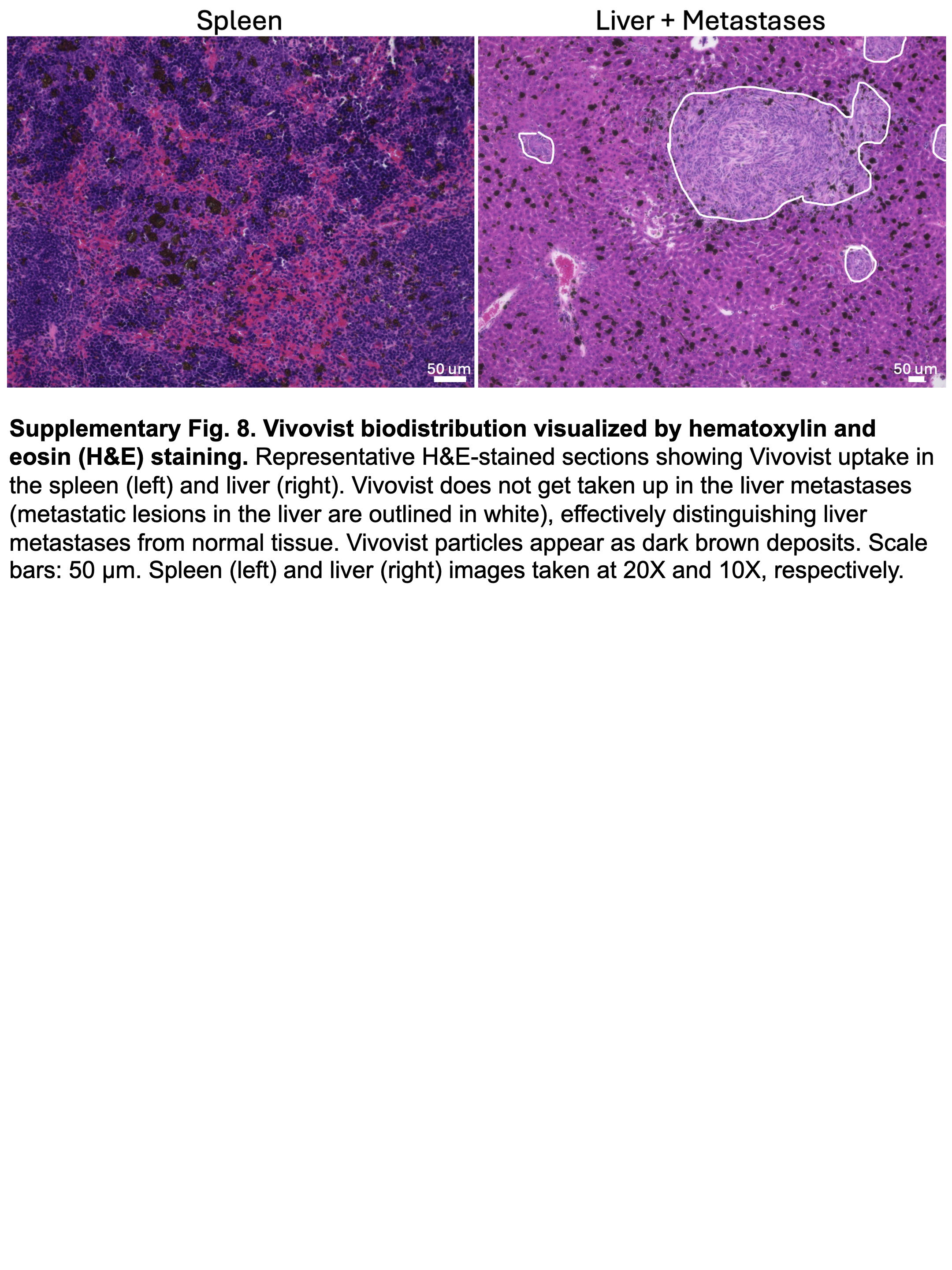

Supplement: Supplementary Figure 8 — Vivovist biodistribution visualized by hematoxylin and eosin (H&E) staining [file crc-25-0414_supplementary_figure_8_suppsf8.png]

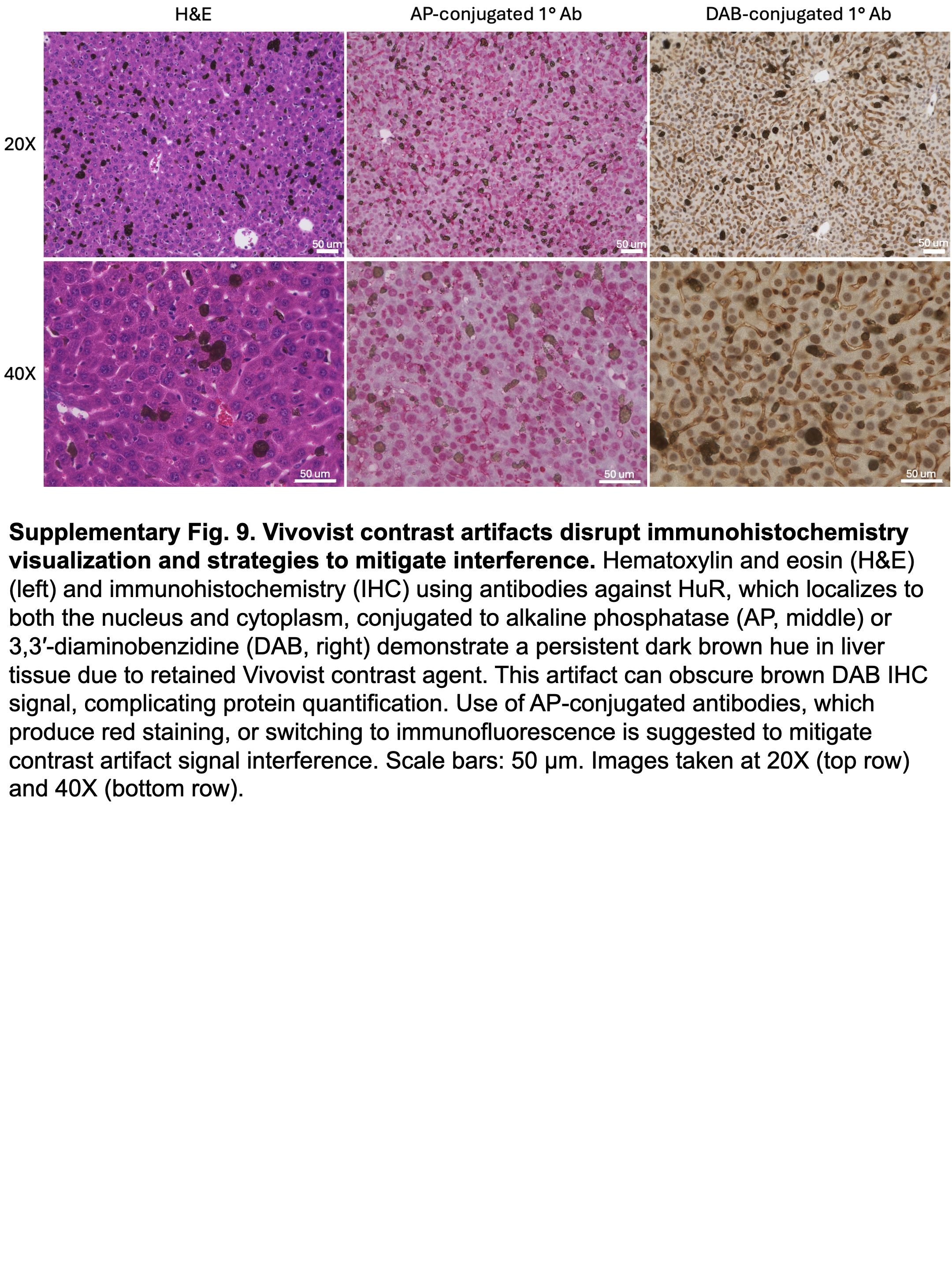

Supplement: Supplementary Figure 9 — Vivovist contrast artifacts disrupt immunohistochemistry visualization and strategies to mitigate interference [file crc-25-0414_supplementary_figure_9_suppsf9.png]

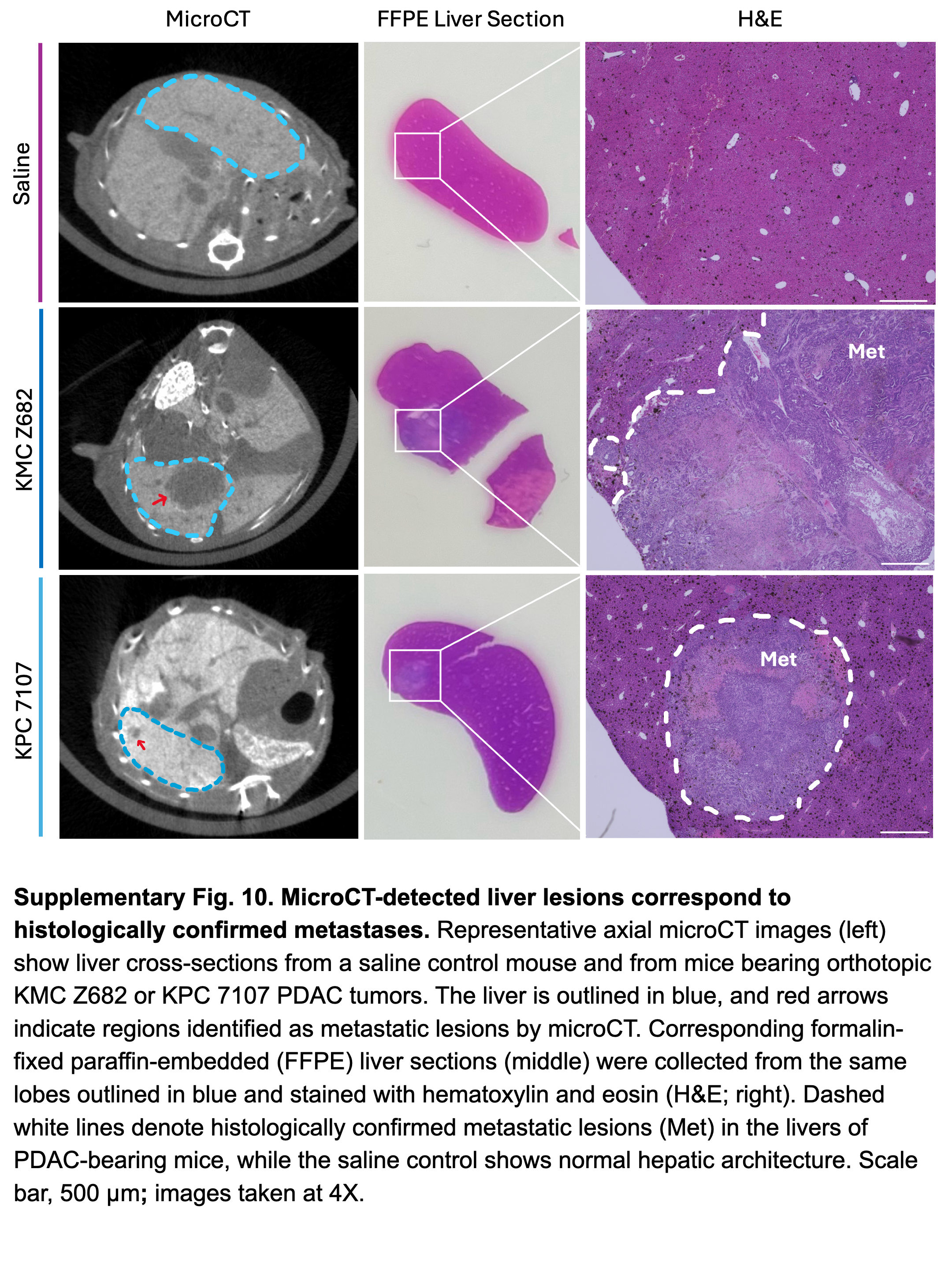

Supplement: Supplementary Figure 10 — MicroCT-detected liver lesions correspond to histologically confirmed metastases [file crc-25-0414_supplementary_figure_10_suppsf10.png]

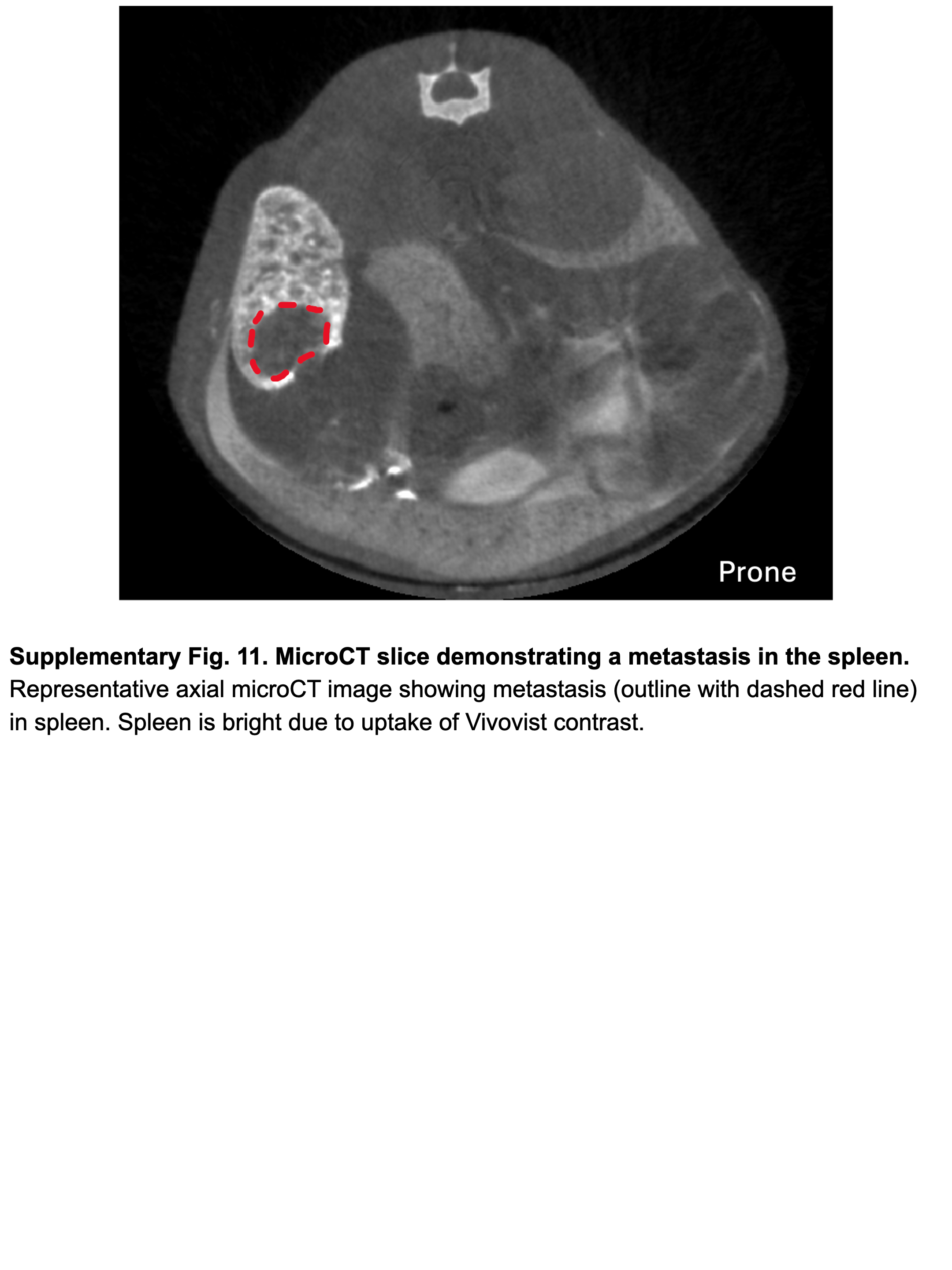

Supplement: Supplementary Figure 11 — MicroCT slice demonstrating a metastasis in the spleen [file crc-25-0414_supplementary_figure_11_suppsf11.png]

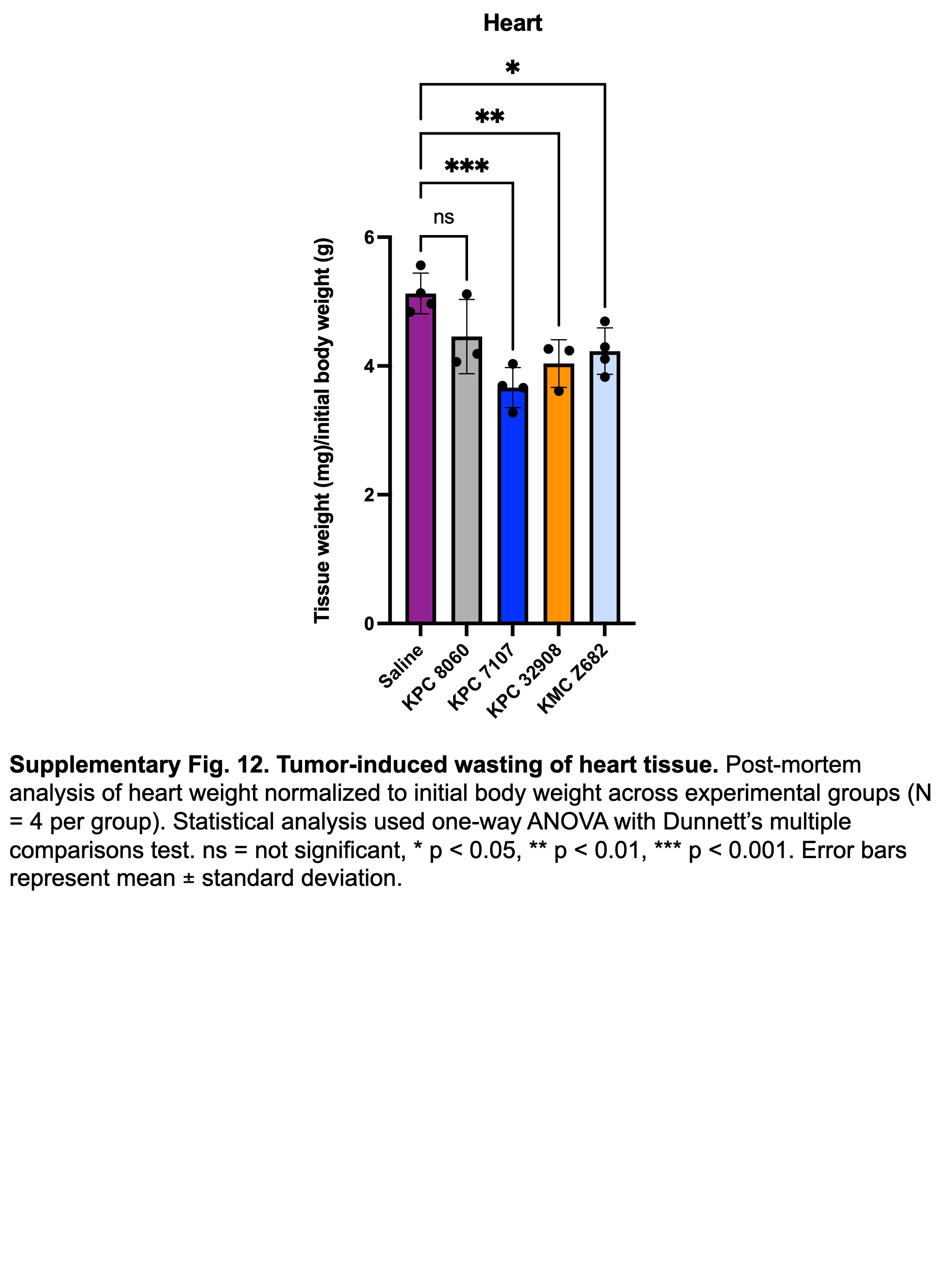

Supplement: Supplementary Figure 12 — Tumor-induced wasting of heart tissue [file crc-25-0414_supplementary_figure_12_suppsf12.png]

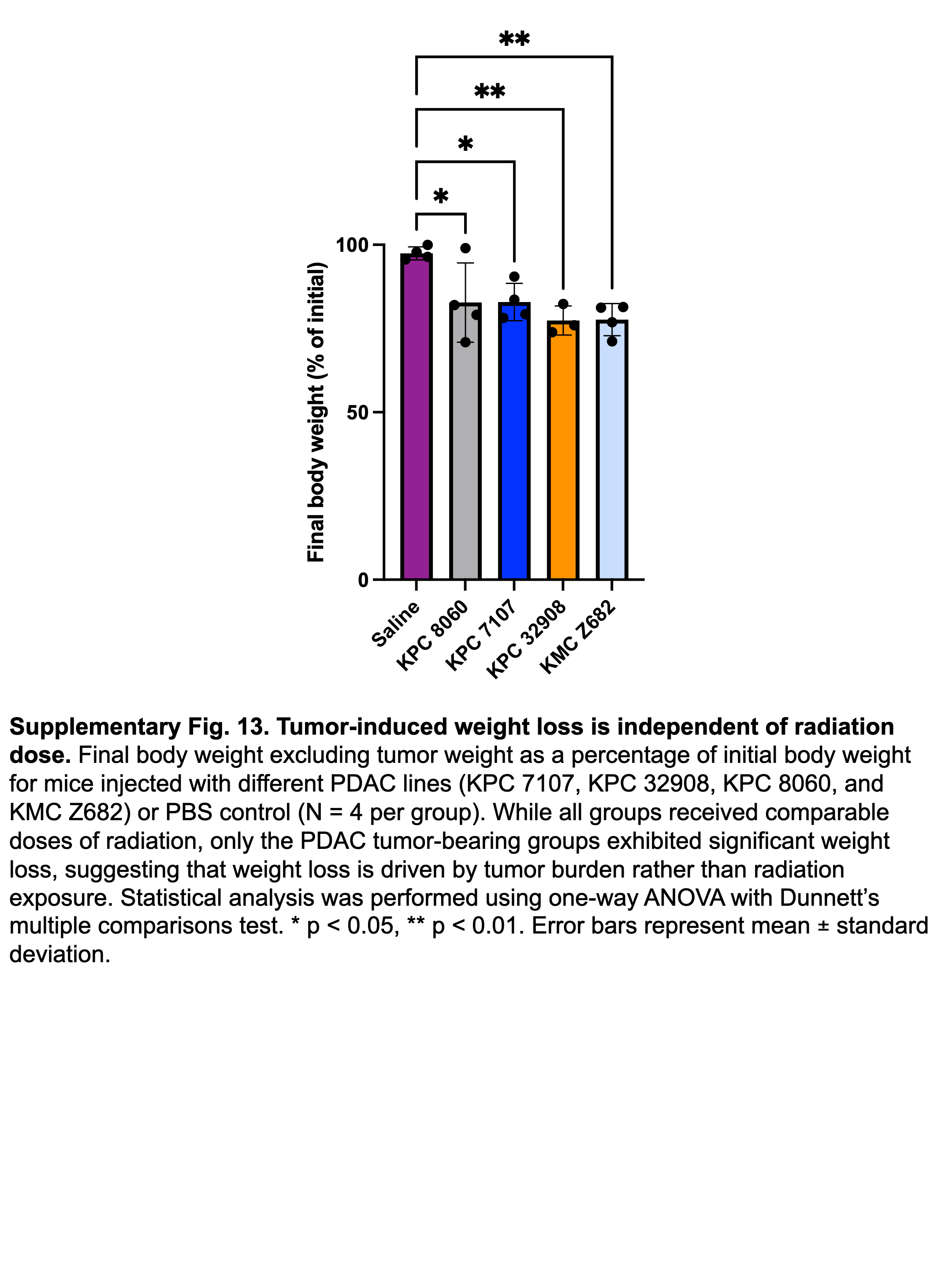

Supplement: Supplementary Figure 13 — Tumor-induced weight loss is independent of radiation dose [file crc-25-0414_supplementary_figure_13_suppsf13.png]

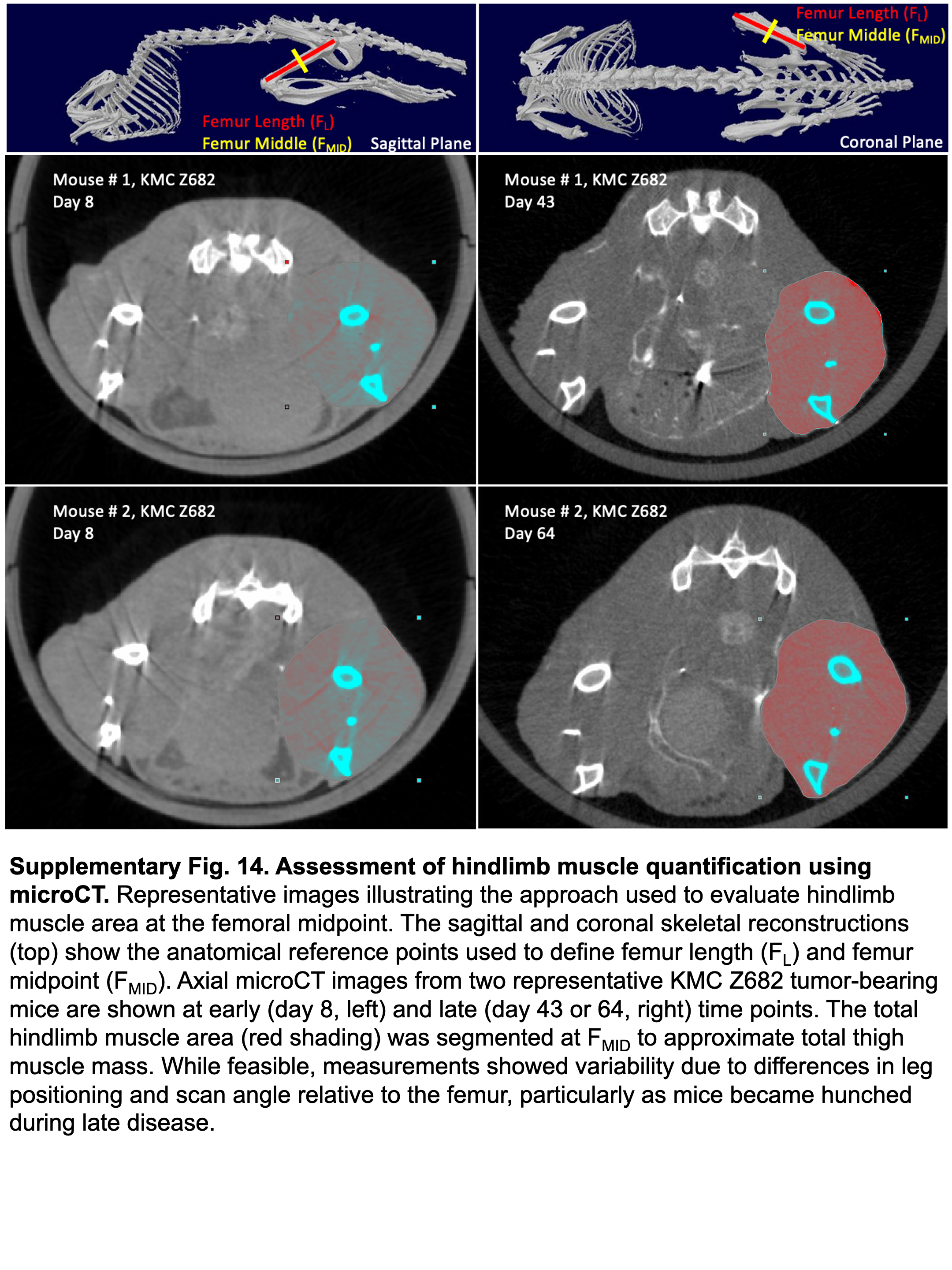

Supplement: Supplementary Figure 14 — Assessment of hindlimb muscle quantification using microCT [file crc-25-0414_supplementary_figure_14_suppsf14.png]

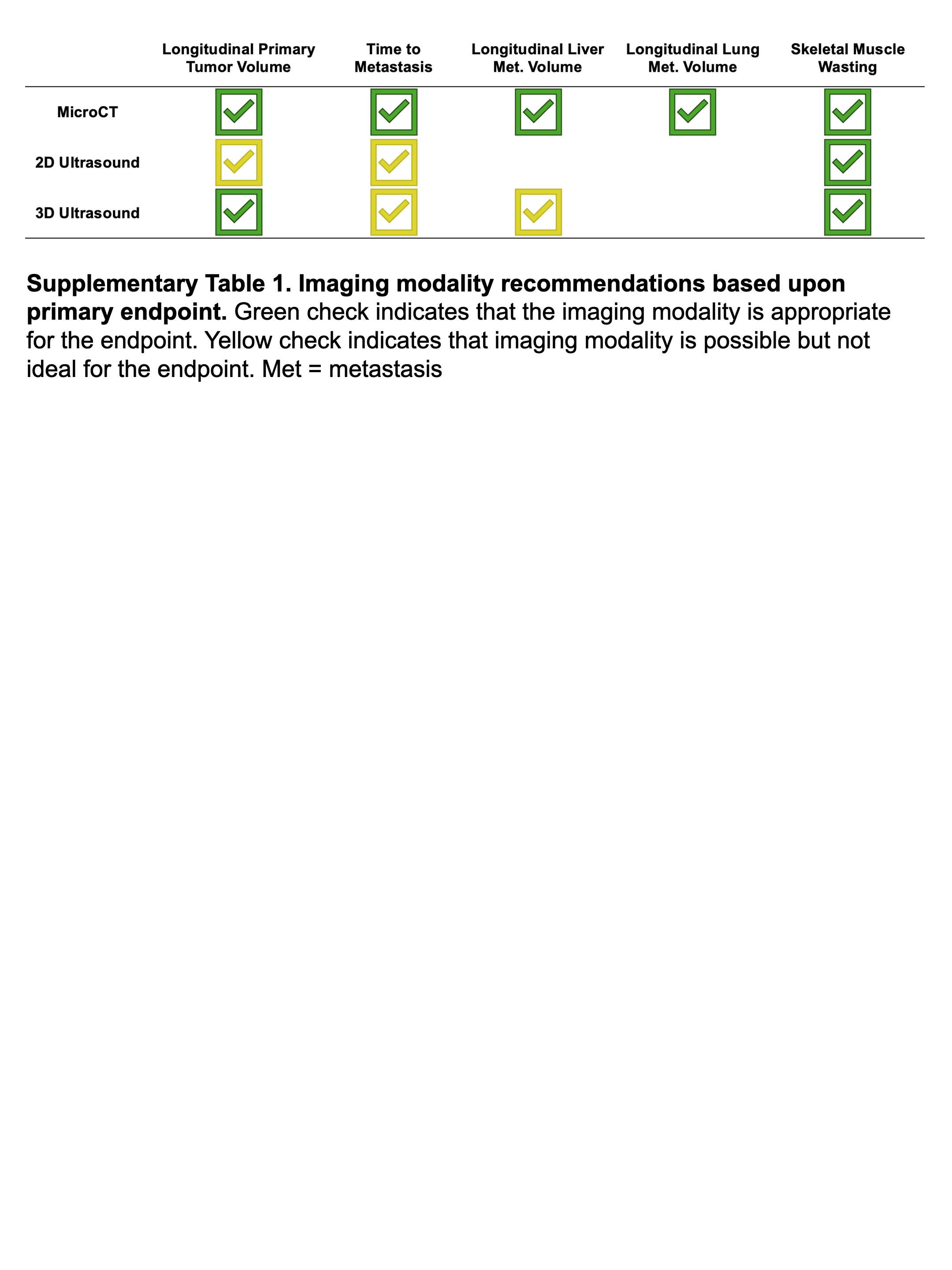

Supplement: Supplementary Table 1 — Imaging modality recommendations based upon primary endpoint [file crc-25-0414_supplementary_table_1_suppst1.png]

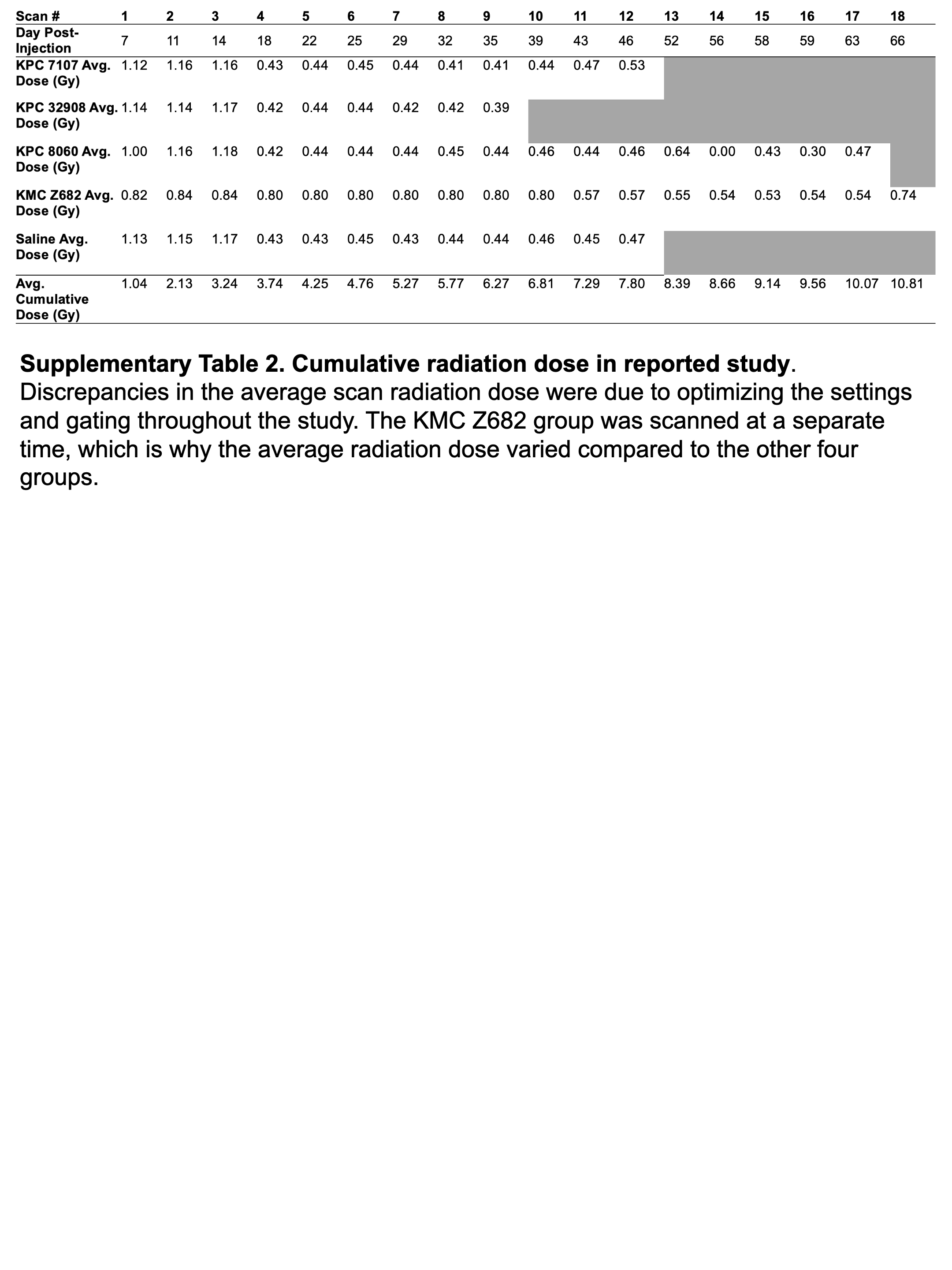

Supplement: Supplementary Table 2 — Cumulative radiation dose in reported study [file crc-25-0414_supplementary_table_2_suppst2.png]
